# Anorexia nervosa in a postoperative patient with Ebstein's anomaly

**DOI:** 10.1002/pcn5.154

**Published:** 2023-11-14

**Authors:** Kengo Sato, Ryosuke Watanabe, Tsuyoshi Okada, Yasushi Nishiyori, Toshiyuki Kobayashi, Shiro Suda

**Affiliations:** ^1^ Department of Psychiatry Jichi Medical University Tochigi Japan

**Keywords:** adult congenital heart disease, anorexia nervosa, Ebstein's anomaly, Fontan procedure

## Abstract

**Background:**

Along with the improved prognosis of patients with congenital heart disease, the associated diverse complications are under scrutiny. Due to various medical restrictions on their upbringing, patients with congenital heart disease often have coexisting mental disorders. However, reports on patients with congenital heart disease and coexisting eating disorders are rare. Here, we report the case of a patient who developed anorexia nervosa (AN) following surgery for Ebstein's anomaly.

**Case Presentation:**

A 21‐year‐old female with Ebstein's anomaly who underwent Fontan surgery was transferred to our institution with suspected AN after >2 years of intermittent stays at a medical hospital for decreased appetite. Initially, she did not desire to lose weight or fear obesity, and we suspected that she was suffering from appetite loss due to a physical condition associated with Fontan circulation. However, the eating disorder pathology gradually became more apparent.

**Conclusion:**

Our experience suggests that patients with congenital heart disease are more likely to have a psychological background and physical problems that might contribute to eating disorders than the general population.

## Graphical abstract

Organic or psychogenic.

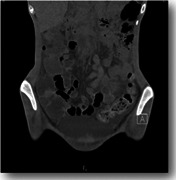

## BACKGROUND

With the development of new medical technologies, approximately 90% of children with congenital heart disease survive into adulthood.^1^, ^2^ However, these patients face problems directly related to the functional status of their heart, such as limited life expectancy, rehospitalization, postoperative sequelae, psychological burdens, and problems related to education, employment, marriage, childbirth, and social security.^3^, ^4^ Medical and social difficulties are likely to be precipitating factors for mental disorders.

In this paper, we report the case of a patient with anorexia nervosa (AN) with Ebstein's anomaly, a severe congenital heart disease, who underwent Fontan surgery. Perhaps because there are few reports on the combination of congenital heart disease and AN, the question of whether the loss of appetite originates from a physical condition or an eating disorder has been debated among clinicians.

To preserve patient anonymity, some details have been slightly altered without affecting our conclusions.

## CASE PRESENTATION

The patient was a 21‐year‐old female during her first visit to our clinic. Her father had major depressive disorder, and her mother had no history of psychiatric disorders. She was the first child of two siblings. She had been diagnosed with Ebstein's anomaly and had undergone a Blalock–Taussig shunting at 1 month of age. At the age of 9 years, she underwent Fontan surgery, and her physical condition improved considerably. After the surgery, she did not require hospitalization until high school graduation, but her parents raised their physically fragile daughter overprotectively. She had never been subjected to any dietary restriction or weight control, but her growth curve almost progressed with a downward deviation of 1 SD. She did not do well at school and tended to have interpersonal problems, but her classmates helped her adjust well.

In April of her 18th year of age, she became employed as an office worker. Her weight was 45 kg and body mass index (BMI) was 18.5 kg/m^2^. Six months later, she was transferred to a new position and became fatigued, experienced frequent stomachaches, and lost her appetite and weight. In February of her 19th year of age, hypotension and hypokalemia were noted, and the patient was referred for admission to the internal medicine ward. She was administered daily intravenous drip infusions, discharged from the hospital at a weight of 40 kg, and returned to work. However, her physical condition worsened due to decreased food intake, and she was readmitted to the hospital. Over the subsequent 2 years, she was hospitalized 14 times in the same way.

**Figure 1:**
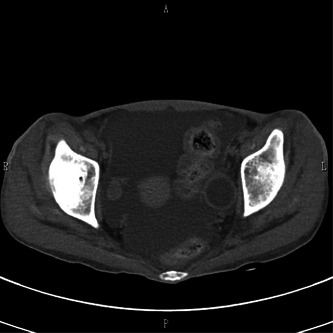
Abdominal computed tomography (transverse view). A large amount of ascites was apparent around the intestine.

**Figure 2:**
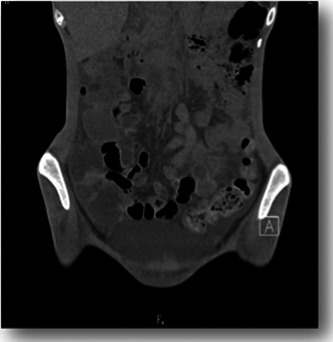
Abdominal computed tomography (longitudinal view). There is a thickening of the intestinal wall that is suggestive of intestinal edema.

In June, at the age of 21 years, because an eating disorder was suspected, she was referred to our department. She stated that she did not want to lose weight, and she weighed 31.9 kg and had a BMI of 13.3 kg/m^2^. Computed tomography (CT) of the head revealed no abnormal findings, and the Wechsler Adult Intelligence Scale, Third Edition, performed in the third month of hospitalization, showed a full‐scale IQ of 74, verbal IQ of 82, and motor IQ of 71. The patient was diagnosed with avoidant/restrictive food intake disorder (ARFID). We started her on a diet of 1150 kcal with 400 kcal of nutritional supplements. After admission, she consumed all meals; however, on the fifth day, she still weighed just 32 kg. She said, “I don't know why my weight isn't increasing.” She began complaining of nausea and gastralgia regularly but rarely vomited. Since her weight did not increase commensurately with her food intake during the first month of admission, we suspected that physical factors related to her heart disease had suppressed her weight gain. Abdominal CT revealed a large amount of ascites and intestinal edema (Figures 1 and 2). There were no significant changes in her blood test results or decreases in protein or albumin levels. According to the pediatric cardiologist, massive ascites and intestinal edema might have originated from a special form of circulation called the Fontan circulation, that is, increased venous pressure of the inferior vena cava. Thus, we considered the possibility that she was unable to consume food because of fear that eating would aggravate her gastrointestinal symptoms. On the 49th day, her weight dropped to 30 kg, and nasogastric tube feeding was required, starting at 1600 kcal. On the 89th day, her weight reached 38.3 kg, and the CT scan that was performed on the 91st day showed that the ascites had almost disappeared. She was able to consume almost 100% of her food orally and was discharged on Day 150. As the patient had a special circulatory condition, the volume of ascites changed depending on the fluctuation of circulating plasma volume and slight changes in nutritional status. Notably, tube feeding improved the patient's nutrition, and her fluid levels normalized.

After 1 month of stable food intake and weight maintenance, she began to vomit after every meal. Her weight decreased drastically, and she was admitted to our hospital for the second time in December. Her weight at admission was 29.4 kg, and her BMI was 12.2 kg/m^2^. Although she initially accepted nasogastric tube feeding well, when we proposed a specific target weight of 40 kg, she expressed resistance to gaining weight. Nevertheless, on the Day 24, her weight recovered to 33 kg, and she expressed a desire to eat orally. Therefore, the nasogastric tube was removed; however, the patient began to eat less frequently. She also told other patients that she had thrown up so much that she had lost too much weight, which indicated that she was deliberately trying to control her weight. Therefore, we revised her diagnosis to AN.

On Day 101, her weight exceeded 39 kg, she began to express a fear of weight gain, and demanded to be discharged from the hospital. As her weight gain was sluggish, at approximately 40 kg, we respected her wishes and discharged her on Day 191. However, soon thereafter, she lost weight and was readmitted to the hospital.

Over the course of 4 years, this patient was out of the hospital for a total of only 99 days. Thus, including the time spent at the previous hospital, she had been hospitalized for more than 6 years. The family cannot hide their anxiety about caring for her at home due to her existing medical and psychiatric disabilities, and they are searching for the next step, which may involve transferring her to a psychiatric hospital, in anticipation of long‐term inpatient care.

## DISCUSSION

Ebstein's anomaly is a congenital heart disease in which one or two valve leaflets of the tricuspid valve are displaced into the right ventricle, resulting in obstruction of tricuspid valve closure and severe regurgitation. The final functional repair procedure is the Fontan surgical techniques, in which venous blood from the body circulation flows directly to the pulmonary artery. With advances in Fontan surgical techniques, the quality of life and prognosis of patients with Ebstein's anomaly have remarkably improved, with a reported survival rate of 94% at 10 years after surgery.^5^ However, Goldberg cautioned that Fontan circulation is an “artificially created chronic heart failure circulation” with high central venous pressure and low cardiac output, which differs from normal circulatory physiology.^6^ Furthermore, although the precise mechanism is unknown, high venous pressure may cause complications that closely relate to dietary intake, such as protein leaks, gastroenteropathy, and liver fibrosis.^7^, ^8^

In the present case, there were no typical symptoms of AN upon admission to our hospital, and ARFID was initially diagnosed. The pathology of AN became apparent only gradually after the second hospitalization. As Nakai et al.^9^ pointed out, there are patients for whom a diagnosis of ARFID was initially made but the pathology of anorexia later became apparent, in parallel with the case of this patient.

To the best of our knowledge, there are no published reports of congenital heart disease coexisting with eating disorders; however, there have been many reports of Turner syndrome, which is associated with a high rate of aortic stenosis concomitant with AN.^10^ Similarly, in the present case, we suspect that the combination of congenital heart disease and eating disorders was not a simple coincidence. Notably, Wray and Sensky^11^ stated that patients with congenital heart disease have significantly delayed social maturation and that one of the triggers of eating disorders is a lack of social contact, due to a dependent lifestyle caused by parental overprotection. In the present case, the patient spent more time in the hospital than at home because of frequent hospitalizations and multistep surgeries since infancy, which resulted in social isolation and insufficient contact with others. The parents devoted their energy to her treatment, but as she still had disabilities, her parents had no choice but to be overprotective. This dependent relationship may have affected her mental development.

Additionally, the protective environment in hospitals may have increased dependency. It is likely that this patient, who was late in maturing socially, was frustrated by the ordeal of finding a job; when she retreated to the hospital, she experienced a familiar and protective environment that she had known since childhood. It has been pointed out that adult patients with congenital heart disease have a high incidence of neurocognitive disorders, which can lead to poor social adjustment.^12^ The intellectual level of the patient in this case was borderline, which may have played a role in her deficiency in social adjustment. Relationships with parents and self‐esteem play a role in the development of AN.^13^, ^14^ The findings related to this patient suggest that difficulties in social maturation in children with congenital heart disease may lead to eating disorders. When this complication occurs, it is difficult to diagnose whether the loss of appetite is due to a physical condition or an eating disorder.

The patient's comments were related to immediate situations or brief emotions and lacked a long‐term perspective. Furthermore, it seemed that she was unable to maintain her weight without physical danger, unless she was in an inpatient environment. Regrettably, she is in a situation where prolonged hospitalization is necessary to avert a physical crisis, and all we can do is hope that one day she will find her way to self‐realization.

## CONCLUSION

We report a case of AN in a postoperative patient with Ebstein's anomaly. A combination of parental protective measures due to congenital heart disease, increased dependence due to prolonged hospitalization, neurocognitive dysfunction due to primary disease, and gastrointestinal symptoms due to Fontan circulation were considered to have resulted in AN as a means of avoiding social participation.

## AUTHOR CONTRIBUTIONS

All authors contributed to the treatment of the patient and the discussion of her symptoms. Kengo Sato wrote the first draft, and all co‐authors approved the final manuscript.

## CONFLICT OF INTEREST STATEMENT

Shiro Suda is the Vice Editor‐in‐Chief of *Psychiatry and Clinical Neurosciences Reports* and a co‐author of this article. They were excluded from editorial decision‐making related to the acceptance and publication of this article. Toshiyuki Kobayashi is an Editorial Board member of *Psychiatry and Clinical Neurosciences Reports* and a co‐author of this article. To minimize bias, they were excluded from all editorial decision‐making related to the acceptance of this article for publication.

## ETHICS APPROVAL STATEMENT

Written informed consent was obtained from the patient and her parents for publication of this case report.

## PATIENT CONSENT STATEMENT

Written informed consent for the presentation of the clinical course was obtained from the patient.

## CLINICAL TRIAL REGISTRATION

N/A.

## Data Availability

N/A.
